# Limited Biomechanical Evidence Behind Single Row Versus Double Row Repair of Subscapularis Tears: A Systematic Review

**DOI:** 10.1016/j.asmr.2022.01.009

**Published:** 2022-03-15

**Authors:** Michelle Xiao, Samuel A. Cohen, Emilie V. Cheung, Seth L. Sherman, Geoffrey D. Abrams, Michael T. Freehill

**Affiliations:** Department of Orthopaedic Surgery, Stanford University School of Medicine, Stanford, California, U.S.A.

## Abstract

**Purpose:**

To systematically review the literature for studies investigating the biomechanical properties of constructs used to repair isolated subscapularis tears in time zero human cadaveric studies.

**Methods:**

A systematic review was performed using Preferred Reporting Items for Systematic Reviews and Meta-Analyses guidelines. Three electronic databases were searched for studies that reported on the construct technique and biomechanical outcomes for the repair of isolated subscapularis tears in human cadaveric specimens. Ultimate load, gap formation, stiffness, and failure mode were documented. Methodological quality was assessed using the Quality Appraisal for Cadaveric Studies (QUACS) scale.

**Results:**

Six articles qualified (104 shoulders [72 single-row, 26 double-row, 6 transosseous]; mean QUACS score 10.5 ± 1) and were analyzed. Studies varied in the number and type of anchors and construct technique (1-2 anchors single-row; 3-4 anchors double-row; bioabsorbable or titanium anchors) and suture(s) used (no. 2 FiberWire or FiberTape), subscapularis tear type (25%, 33%, 50%, or 100% tear), and whether a knotless or knotted fixation was used. In studies that created full-thickness, upper subscapularis tears (Fox-Romeo II/III or Lafosse II), no significant differences were seen in ultimate load, gap formation, and stiffness for knotted versus knotless single-row repair (2 studies) and single-row versus double-row repair (1 study). Double-row repair of complete subscapularis tears demonstrated higher ultimate load, stiffness, and lower gap formation in 1 study. Ultimate load differed between the studies and constructs (single-row: range, 244 N to 678 N; double-row: range 332 N to 508 N, transosseous: 453 N). Suture cutout was the most common mode of failure (59%).

**Conclusion:**

Because of the limited number of studies and varying study designs in examining the biomechanical properties of repair constructs used for subscapularis tears, there is inconclusive evidence to determine which construct type is superior for repairing subscapularis tears.

**Clinical Relevance:**

Results from biomechanical studies of clinically relevant subscapularis repair constructs are important to guide decision-making for choosing the optimal construct for patients with subscapularis tears.

The subscapularis is the largest and most powerful rotator cuff muscle. It primarily functions as an internal rotator of the humerus but also plays an important role in balancing glenohumeral joint forces and in maintaining anterior shoulder stability.^1^, ^2^, ^3^ Historically, the prevalence of subscapularis tears was thought to be low and reported in less than 5% of rotator cuff tears.^4^ However, with the progression of shoulder arthroscopy techniques and advanced imaging, it is now estimated that 19% to 59% of rotator cuff tears also have combined subscapularis involvement.^1^^,^^5^, ^6^, ^7^, ^8^, ^9^ Isolated subscapularis tears remain rare, accounting for around 5% of all rotator cuff repairs.^5^^,^^8^^,^^10^ These isolated tears are typically the result of a traumatic injury and occur more commonly in younger patients.^3^^,^^11^^,^^12^

Adequate fixation of subscapularis tears is important for restoring normal joint function.^13^ Open repair of the subscapularis was once considered the gold standard,^14^ but arthroscopic, single- or double-row, suture anchor constructs have become increasingly used.^15^, ^16^, ^17^, ^18^, ^19^, ^20^

Clinical studies have demonstrated that arthroscopic single- and double-row repair of subscapularis tears results in significant improvements in clinical outcomes.^21^ Numerous biomechanical studies have compared the strength of single-row and double-row repairs for supraspinatus and infraspinatus tears,^15^, ^16^, ^17^, ^18^, ^19^, ^20^^,^^22^ and a systematic review concluded that double-row constructs restored more of the anatomic footprint and had stronger biomechanical properties compared to single-row repairs for posterosuperior rotator cuff tears.^23^ However, the anatomical and functional differences between the subscapularis and the posterosuperior rotator cuff necessitate biomechanical studies pertaining directly to the subscapularis to determine the strongest repair construct.^24^

Given that there is limited tendon-to-bone healing in the early postoperative window, the initial success of the repair depends largely on the strength of the construct and its ability to transfer load from tendon to bone.^20^ Thus results from biomechanical studies of clinically relevant subscapularis repair constructs are important to guide decision-making for choosing the optimal construct for patients with subscapularis tears. The purpose of this study was to systematically review the literature for studies investigating the biomechanical properties of constructs used to repair isolated subscapularis tears in time zero human cadaveric studies.

## Methods

### Literature Review and Search Strategy

This systematic review was performed using Preferred Reporting Items for Systematic Reviews and Meta-Analyses guidelines.^25^ Two authors conducted separate searches of the following medical databases: PubMed, SCOPUS, and Cochrane Central Register of Controlled Trials. The searches were performed on December 22, 2021, and confirmed by the senior author (M.T.F.). The search string used was as follows: subscap∗[All Fields] AND (construct[tw] OR strength[tw] OR fixation[tw] OR repair[tw] OR tear[tw] OR reconstruct∗[tw] OR fail∗[tw]) AND (biomech∗[tw] OR cadav∗). Articles published from inception to December 22, 2021 were included for screening.

### Eligibility Criteria

**Fig 1:**
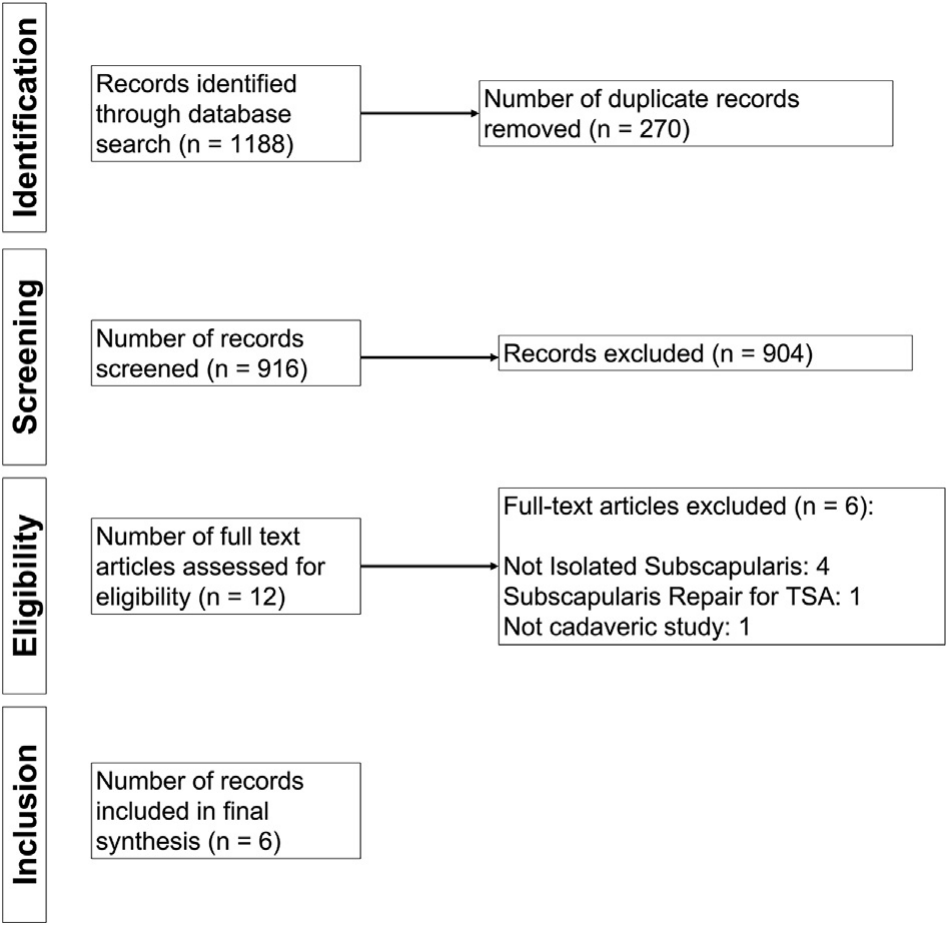
Flow diagram summarizing the literature search, screening, and review.

Eligible studies consisted of biomechanical studies published in the English language that reported on the construct technique and biomechanical outcomes for the repair of simulated isolated subscapularis tears in human cadaveric specimen. Exclusion criteria were (1) review articles, animal studies, clinical studies, book chapters, technique articles, and case reports; (2) studies pertaining to subscapularis repair during shoulder arthroplasty; and (3) studies examining tendon transfers. In the event of different studies with duplicate (or overlapping) subject populations, the study with the greatest number of subjects or greatest clarity of methods and results was included if the subjects could not be separated. After removal of duplicates, titles or abstracts were screened, and full text articles were further assessed based on inclusion and exclusion criteria by 2 independent reviewers (M.X., S.A.C.). The search results were reviewed for duplicates and the inclusion criteria to determine the articles that were included in the final analysis (Fig 1).

### Data Extraction

Articles were reviewed and data were extracted from the included studies by the 2 independent reviewers using the methodology recommended by Harris et al.^26^ All study, specimen, and biomechanical parameters were collected. Parameters analyzed included year of publication, number of specimens, number of shoulders, mean age, subscapularis tear size, construct type, cyclic loading protocol, ultimate load, gap formation, stiffness, failure mode, and repair footprint contact area. Extracted data were cross-checked for accuracy by the two reviewers and recorded onto a shared spreadsheet.

### Quality Assessment

The risk of study bias and methodological quality was assessed using the Quality Appraisal for Cadaveric Studies (QUACS) scale, a 13-item checklist that assesses the design, conduct, and report of cadaveric dissection studies for inclusion into systematic reviews.^27^ Scores are reported as poor (<20%), fair (>20% and <40%), moderate (>40% and <60%), substantial (>60% and <80%), or excellent (>80%).

### Statistical Analysis

Due to the heterogeneity between studies, pooling of data and meta-analysis was not performed. Thus, a qualitative synthesis with descriptive summaries of the studies is presented.

## Results

### Characteristics of Included Studies

The initial search yielded 1188 articles. After removing duplicates, 916 records were screened for eligibility. Of these, 12 articles underwent full text review, resulting in six articles that were included and analyzed (Fig 1).^28^, ^29^, ^30^ According to the QUACS, three studies were excellent (>11/13),^28^^,^^31^^,^^32^ and 3 studies were of substantial (9/13 or 10/13) quality.^29^^,^^30^ The number of specimens per study ranged from 6 to 18 for a total of 63 specimens and 104 shoulders included in this review. The mean age of the cadaveric specimens ranged from 62.4 to 78 years. The average native subscapularis footprint area was reported in three studies,^28^^,^^30^^,^^31^ and it ranged from 295 mm^2^ to 631.5 mm^2^ (Table 1).

**Table 1:** Characteristics of Included Studies

Study	Journal	QUACS	Number of Specimens	Number of Shoulders	Specimen Age, (y), Mean ± SD (Range)	Native Footprint Size (mm^2^), Mean ± SD (Range)
Borbas 2021	KSSTA	11	18	18	78 ± 8	631.5 ± 131.2
Dyrna 2019	*Arthroscopy*	11	15	30	62.4 (58-74)	486.9 ± 59.7
Lorbach 2016	KSSTA	9	6	12	NS	NS
Sgroi 2021	CORR	12	8	16	69 (61-75)	NS
Wellmann 2009	KSSTA	10	10	16	65.4 ± 13	NS
Wheeler 2010	*Arthroscopy*	10	6	12	68 ± 12	295 (237-365)

QUACS, Quality Appraisal for Cadaveric Studies; SD, standard deviation; KSSTA, *Knee Surgery, Sports Traumatology, Arthroscopy*; CORR, *Clinical Orthopaedics and Related Research*; NS, not specified.

### Subscapularis Repair Constructs

Three studies^28^^,^^32^^,^^33^ used the Fox-Romeo^34^ classification system to recreate type II (complete tear of the upper 25%) and III (complete tear of the upper 50%) tears of the subscapularis, 1 study^31^ used the Lafosse classification^8^ to recreate type II tears (complete tear of the upper 1/3), and 2 studies created complete full-thickness tears^29^^,^^30^ (Table 2). Of the included studies, two^28^^,^^29^ compared single-row and double-row repair, one^30^ compared single-row to transosseous repair, and 3 studies^31^, ^32^, ^33^ used only single-row suture anchor constructs. Two studies compared knotted and knotless single-row repairs.^31^^,^^32^ The 4 studies^28^^,^^31^, ^32^, ^33^ published most recently used bioabsorbable suture anchors, whereas titanium suture anchors were used in the other 2 studies (Table 2).^29^^,^^30^

**Table 2:** Summary of Subscapularis Repair Construct Types in the Included Studies

Study	Tear Type	Repair	SR Stitch	SR Anchors	DR/TO Stitch	DR Anchors	Suture
Borbas 2021	Lafosse II	SR	1. Knotted Lasso-loop mattress 2. Knotted horizontal Mattress 3. Knotless FiberTape	DL 5.5 mm PEEK or 4.75 mm Swivelock (1 anchor)	N/A	N/A	NS or FiberTape
Dyrna 2019	Fox-Romeo II and III	SR vs HDR vs DR	Simple mattress	DL 4.5 mm Bio-Corkscrew (2 anchors)	Medial: knotless; Lateral: knotless	SL medial and DL lateral 4.75-mm absorbable SwiveLock (3 anchors)	SR: No. 2 FiberWire; DR: FiberTape
Lorbach 2016	Fox-Romeo II and III	SR	Double mattress	DL 5.5 mm Bio-Corkscrew (1 anchor)	N/A	N/A	No. 2 FiberWire
Sgroi 2021	Fox-Romeo III	SR	1. Knotted (Modified MA) 2. Knotless FiberTape	5.5 mm Bio-Corkscrew or 5.5 mm Swivelock (1 anchor)	N/A	N/A	No. 2 FiberWire or FiberTape
Wellmann 2009	Full-Thickness Complete Tear	SR vs DR	Modified MA	DL 5.5 mm titanium Corkscrew (2 anchors)	Medial: horizontal mattress; Lateral: knotless	Medial: SL 5.0-mm titanium Corkscrew (2 anchors); Lateral: DL 4.5-mm Bio-PushLock (2 anchors)	No. 2 FiberWire
Wheeler 2010	Full-Thickness Complete Tear	SR vs TO	Horizontal mattress	DL 5.0 mm titanium Corkscrew (2 anchors)	Modified MA	3 bone tunnels	No. 2 FiberWire

SR, single row; DR, double row; TO, transosseous; HDR, hybrid double row; DL, double-loaded; SL, single-loaded; MA, Mason Allen; N/A, not applicable.

Double-row repairs used three^28^ or four^29^ suture anchors. The double-row, knotless construct in the study by Dyrna et al.^28^ placed 1 lateral row suture anchor out of the native subscapularis footprint in a superolateral position close to the entrance of the bicipital groove. Wellman et al.^29^ repaired the subscapularis tendon using a double row suture-bridge technique, with a modified Mason-Allen stitch used to tie the medial row. The authors positioned the 2 lateral row anchors just medial to the bicipital groove and the 2 medial row anchors 10 to 12 mm medially to the lateral row anchors. Wheeler et al.^30^ used an open transosseous construct for subscapularis repair with 3 bone tunnels made at the lesser tuberosity. Three modified Mason-Allen stitches were used to secure the subscapularis repair (Table 2).

### Biomechanical Properties

All 6 studies recorded ultimate load and mode of failure. The load to failure protocol for the studies was either 0.5 mm/s or 1 mm/s. The mean ultimate load for single-row constructs ranged from 244 N to 678 N. No significant differences in ultimate load were seen in the 2 studies comparing knotted and knotless single-row constructs.^29^^,^^30^ The mean ultimate loads for the 2 studies with double-row constructs were 332 N and 508 N,^28^^,^^29^ and the ultimate load for transosseous repair was 453 N. One study^29^ found that double-row repair resulted in a significantly higher ultimate load compared to single-row repair of complete subscapularis tears, whereas another study^28^ did not find a significant difference in ultimate failure load between single-row and double-row constructs of full-thickness partial subscapularis tears. Lorbach et al.^33^ reported no significant difference in ultimate failure load between Fox-Romeo type II and III tears repaired with a single-row construct. Overall, suture cutout through the tendon was the most common mode of failure (59%; Table 3).

**Table 3:** Summary of Biomechanical Results of the Included Studies

Study	Cyclic Loading	Gap Formation (mm)	Ultimate Load (N)	Footprint Contact Area (%)	Stiffness (N/mm)	Failure Mode	Failure Loading
Borbas 2021	Preload 10N; 10-100 N for 300 cycles at 2 mm/s	SR lasso: 1.3 ± 0.5 SR mattress: 1.3 ± 0.5 SR tape: 1.1 ± 0.9	SR lasso: 630.8 ± 145.3 SR mattress: 586.9 ± 220.7 SR tape: 678.2 ± 236.5	SR lasso: 65.4 ± 10.2 SR mattress: 66.8 ± 9 SR tape: 62.3 ± 9.7	SR lasso: 88 ± 30.3 SR mattress: 65 ± 27 SR tape: 83.9 ± 32.9	Suture cutout (8/18); proximal humerus fracture (5/18); MT junction (4/18); lesser tuberosity avulsion (1/18)	0.5 mm/s
Dyrna 2019	Preload 10 N; 10-100 N for 300 cycles at 0.5 Hz	SR Type II: 1.4 ± 0.5 HDR Type II: 1.3 ± 0.5 SR Type III: 1.8 ± 0.6 HDR Type III: 1.4 ± 0.5 DR Type III:1.5 ± 0.5	SR Type II: 531 ± 129 HDR Type II: 451 ± 132 SR Type III: 451 ± 124 HDR Type III: 548 ± 228 DR Type III: 508 ± 170	SR Type II: 88.4 ± 8.9 HDR Type II: 95.1 ± 7.9 SR Type III: 73.6 ± 10.9 HDR Type III: 84.4 ± 9.4 DR Type III: 84.1 ± 12.3	SR Type II: 36.5 ± 8.7 HDR Type II: 36.4 ± 6.9 SR Type III: 36.2 ± 5.5 HDR Type III: 48.3 ± 11.5 DR Type III: 42.3 ± 12.1	Suture cutout (16/30), Anchor pullout (7/30), lesser tuberosity fracture (4/30), medial tendon failure (3/30)	0.5 mm/s
Lorbach 2016	Preload 10 N; 10-60 N for 50 cycles Stepwise increase to 100 N and 180 N for 50 cycles	At 100 N: SR Type II: 5.1 mm SR Type III: 4.3 mm	SR Type II: 486 ± 167 SR Type III: 455 ± 213	NS	NS	SR Type II: Anchor pullout or bone fracture (6/6) SR Type III: Anchor pullout (2/6), Suture cutout (4/6)	NS
Sgroi 2021	Preload 10N; 10-60 N for 50 cycles Stepwise increase to 100 N and 180 N for 50 cycles at 1 Hz	No differences between groups	SR knotted: 521.1 ± 266.2 SR knotless: 475.8 ± 183.3	NS	At 10-100 N: SR knotted: 45.0 ± 4.8 SR knotless: 45.2 ± 6	SR knotted: anchor pullout (2/8); suture cutout (6/8) SR knotless: anchor pullout (2/8); suture cutout (3/8); suture slipped out of eyelet (3/8)	1 mm/s
Wellmann 2009	5-100 N for 100 cycles	SR: 1.7 ± 0.5 DR: 1.2 ± 0.3∗	SR: 244 ± 40N DR: 332 ± 39N∗	NS	SR: 55 ± 8 DR: 81 ± 12∗	SR: Suture cutout (7/8); lesser tuberosity fracture (1/8) DR: Suture cutout (8/8)	1 mm/s
Wheeler 2010	Preload 60 N; 76-183 N for 50 cycles at 0.1 Hz	SR: 2.38 ± 1.6 TO: 0.64 ± 0.4∗	SR: 392.6 ± 78 TO: 453.2 ± 66	SR: 65.9 ± 27.9 TO: 94.2 ± 37.4	NR	SR: suture cutout (3/6), anchor pullout (3/6); TO: suture cutout (6/6)	0.5 mm/s

SR, single row; DR, double row; TO, transosseous; MT, musculotendinous; NS, not specified; N, Newtons; Hz, Hertz.

∗ Statistically significant difference between groups.

The number of cycles of loading ranged from 50 to 300 (Table 3). All articles used a force controlled cyclic loading protocol to assess gap formation. There were no significant differences in gap formation in studies comparing knotted and knotless single-row constructs. Two studies^29^^,^^30^ found that double-row or transosseous constructs resulted in significantly less gap formation compared to single-row repair of complete subscapularis tears, whereas one study^28^ found no difference between constructs in gap formation after repair of Fox-Romeo type II and III tears. Wellmann et al.^29^ reported that the double-row technique resulted in a stiffer construct compared to single-row repair, although Dyrna et al.^28^ found no significant difference in stiffness and pressurized contact area between single- and double-row repairs. Wheeler et al.^30^ reported no significant differences in contact area between single-row and transosseous repair (Table 3).

## Discussion

The present systematic review included 6 studies that investigated the biomechanical properties of repair constructs used for isolated subscapularis tears in cadaveric specimens. Despite the variation in the number of anchors, anchor type, suture type, and tear pattern between the studies, all 6 articles investigated single-row repair constructs. Other repair types that were studied included double-row and transosseous repair. Although tendon healing is affected by a variety of parameters such as patient demographics, tear characterization, biological factors, and tissue quality, biomechanical properties of the repair construct are strong predictors of successful repair.^35^, ^36^, ^37^ These biomechanical properties include low gap formation, high ultimate load, large footprint contact area, and higher stiffness, which are especially important during the initial phase of postoperative period, when biological healing results in a structurally and mechanically weakened tendon-to-bone interface.^37^, ^38^ In studies that created full-thickness, upper subscapularis tears (Fox-Romeo II/III or Lafosse II), no significant differences were seen in ultimate load, gap formation, and stiffness for knotted compared to knotless single-row repair. In studies that compared single-row to double-row repair for subscapularis tears, similar biomechanical properties were reported for single-row and double-row repair of Fox-Romeo II/III tears, but a higher ultimate load, lower gap formation, and higher stiffness was found for double-row repair of complete subscapularis tears.

Single-row repair in the current review used either 1 or 2 double-loaded suture anchors and were knotless or tied with variations of mattress or modified Mason-Allen stitches. Sgroi et al.^32^ compared knotted and knotless single-row repair for Fox-Romeo III subscapularis tears and found no significant differences in biomechanical properties for both constructs. Likewise, Borbas et al.^31^ compared 3 different single-row constructs (knotted lasso-loop mattress, knotted mattress, and knotless suture tape) for Lafosse II subscapularis tears and found that the constructs resulted in similar ultimate load, gap formation, and stiffness. The authors did report a significantly higher pressurized footprint coverage for the knotted lasso-loop mattress and knotless construct compared to the knotted mattress repair. Overall, single row repair accounted for the most common construct investigated in biomechanical studies of subscapularis repair, which is in line with findings that the majority of clinical studies use single row constructs as well.^21^

There were 2 studies that directly compared single-row to double-row suture anchor repair, although the double-row constructs varied between studies. Dyrna et al.^28^ focused their repair toward the “leading-edge” by placing a lateral-row anchor superolaterally outside of the native footprint. Wellmann et al.^29^ used a suture bridge construct, which is designed to maximize the pressurized contact area while preserving tendon vascularity.^37^ These studies also conflicted with regards to whether there was a biomechanical difference between the two construct types. Although Dyrna et al.^28^ found no difference in load to failure between single- and double-row repairs, Wellmann et al.^29^ reported significantly higher ultimate load and stiffness for double-row constructs. These variations in results are likely due to the differences in study design, because the study by Dyrna and colleagues^28^ was conducted more recently, included only Fox-Romeo II and III tears, and repaired tears using absorbable suture anchors and a knotless double-row configuration with FiberTape. On the other hand, Wellmann et al.^29^ repaired complete subscapularis tears with titanium suture anchors, and their double-row construct used a tied medial row. From these studies, tear size may play an important role in choosing a repair construct. For smaller tears, no biomechanical differences may be seen between single-and double-row repair, but for larger, complete tears, double-row constructs may be more biomechanically favorable. These findings are similar to those in clinical practice, as clinical studies on subscapularis repair tended to use double-row constructs for larger, Lafosse III and IV tears, and single-row constructs for smaller subscapularis tears.^21^

Prior systematic reviews of biomechanical evidence for rotator cuff constructs have focused on the posterosuperior rotator cuff, concluding that double-row constructs are biomechanically stronger to single-row fixation.^23^^,^^39^ Shi et al.^39^ performed a meta-regression of 40 posterosuperior rotator cuff biomechanical studies and found that the repair material such as number of sutures, suture limbs passed through the tendon, and mattress stitches are stronger predictors of rotator cuff repair strength than the type of construct. Clinically, Chen et al.^40^ found that after meta-analysis, double-row repairs resulted in improved tendon healing compared to single-row fixation for patients with larger posterosuperior rotator cuff tears (>3 cm), but the authors did not find clinically significant differences in patient-reported outcomes. Many of the arthroscopic constructs currently used for subscapularis repair are derived from repair techniques used for supraspinatus tears. Yet the subscapularis has the largest anatomic footprint out of all rotator cuff tendons and differs in stiffness and strength,^41^, ^42^, ^43^ necessitating separate biomechanical and clinical studies. Despite the importance of the subscapularis, there is a relative paucity of studies in the literature in comparison to that of posterior superior tears.^3^^,^^44^ The small number of studies found in the current investigation mirror the finding that fewer clinical studies targeting subscapularis repair exist as compared to studies of the posterosuperior rotator cuff.^21^^,^^45^

Isolated tears make up about 10% to 25%^8^^,^^46^ of all subscapularis lesions (5% of all rotator cuff repairs)^5^^,^^8^^,^^10^ and are usually associated with a traumatic event, although subscapularis tears occur more often in combination with other rotator cuff tears. The rate of isolated subscapularis re-tear has been reported to range from 5% to 17%.^8^^,^^46^, ^47^, ^48^, ^49^, ^50^ Yoon et al.^46^ compared arthroscopic single- versus double-row suture bridge repair for isolated subscapularis tears and found no differences in outcomes or re-tear rates. In this review, the most common failure mode for all repair types was suture cutout through the tendon, although differences in failure mode between construct types could not be determined. Additionally, the increased cost, operating room time, and technical difficulty of double-row compared to single-row repair may influence construct choice. In cases of smaller tears that require less fixation strength, single-row constructs may be adequate.

### Limitations

This analysis has several limitations. The study design resulted in analysis of relatively few studies. The studies included in this review varied with respect to subscapularis tear size, anchor placement, and testing parameters. Given the varying parameters, head-to-head comparisons of knotted and knotless constructs or single- and double-row constructs yielded few studies, which may affect final conclusions. Because of the study heterogeneity, pooling of results could not be performed. The double-row constructs included in this review were either all-knotless^28^ or tied medial row.^29^ As such, no support for or against the tying of the medial row can be determined. Additionally, 1 study investigating transosseous repair was included in this review, although this repair construct is less frequently used. All studies included in this review were cadaveric studies that only assessed time zero biomechanical properties. Thus healing responses, tissue quality, and forces applied are different than in vivo.^51^ Few studies included in this review were homogenous and compared similar groups. Thus conclusions with regard to whether single-row versus double-row repair of subscapularis tears is biomechanically favorable cannot be determined. Finally, the loading protocols and construct materials such as suture and anchor type differed among all studies, supporting the possibility that the biomechanical differences detected in this study may be due to differences in study design.

## Conclusions

Because of the limited number of studies and varying study designs in examining the biomechanical properties of repair constructs used for subscapularis tears, there is inconclusive evidence to determine which construct type is superior for repairing subscapularis tears.

## Supplementary Data

ICMJE author disclosure forms
